# Trends and patterns of initial percutaneous nephrolithotomy and subsequent procedures among commercially-insured US adults with urinary system stone disease: a 10-year population-based study

**DOI:** 10.1007/s00345-022-04210-0

**Published:** 2022-11-19

**Authors:** Stephen S. Johnston, Barbara H. Johnson, Pragya Rai, Philippe Grange, Tony Amos, Sudip Ghosh, Noor Buchholz

**Affiliations:** 1 Epidemiology, Medical Devices, Johnson & Johnson, 410 George Street, New Brunswick, NJ 08901 USA; 2 Medical Affairs, Ethicon, Cincinnati, OH USA; 3 Health Economics and Market Access, Ethicon, Raritan, NJ USA; 4 Scientific Office, U-Merge Ltd. (Urology for Emerging Countries), London, UK

**Keywords:** Incidence, Nephrolithiasis, Percutaneous nephrolithotomy, Urolithiasis

## Abstract

**Purpose:**

To describe trends and patterns of initial percutaneous nephrolithotomy (PCNL) and subsequent procedures from 2010 to 2019 among commercially-insured US adults with urinary system stone disease (USSD).

**Methods:**

Retrospective study of administrative data from the IBM® MarketScan^®^ Database. Eligible patients were aged 18–64 years and underwent PCNL between 1/1/2010 and 12/31/2019. Measures of interest for analysis of trends and patterns included the setting of initial PCNL (inpatient vs. outpatient), percutaneous access (1 vs. 2-step), and the incidence, time course, and type of subsequent procedures (extracorporeal shockwave lithotripsy [SWL], ureteroscopy [URS], and/or PCNL) performed up-to 3 years after initial PCNL.

**Results:**

A total of 8,348 patients met the study eligibility criteria. During the study period, there was a substantial shift in the setting of initial PCNL, from 59.9% being inpatient in 2010 to 85.3% being outpatient by 2019 (*P* < 0.001). The proportion of 1 vs. 2-step initial PCNL fluctuated over time, with a low of 15.1% in 2016 and a high of 22.0% in 2019 but showed no consistent yearly trend (*P* = 0.137). The Kaplan–Meier estimated probability of subsequent procedures following initial PCNL was 20% at 30 days, 28% at 90 days, and 50% at 3 years, with slight fluctuations by initial PCNL year. From 2010 to 2019, the proportion of subsequent procedures accounted for by URS increased substantially (from 30.8 to 51.8%), whereas SWL decreased substantially (from 39.5 to 14.7%) (*P* < 0.001).

**Conclusions:**

From 2010 to 2019, PCNL procedures largely shifted to the outpatient setting. Subsequent procedures after initial PCNL were common, with most occurring within 90 days. URS has become the most commonly-used subsequent procedure type.

**Supplementary Information:**

The online version contains supplementary material available at 10.1007/s00345-022-04210-0.

## Introduction

The incidence of urinary system stone disease (USSD), which includes stones in kidneys and/or ureter, has been on the rise over the past three decade [1, 2]. The surgical management of USSD has also evolved over the years, from open surgical lithotomy to minimally invasive endourological approaches, namely, extracorporeal shockwave lithotripsy (SWL), ureteroscopy with laser lithotripsy (URS), and percutaneous nephrolithotomy (PCNL) [3, 4]. Of these three procedures, PCNL is the most complex and recommended by the American Urological Association for removal of stones > 2 cm, but may also be used for stones ≤ 2 cm [5]. Additional procedures after PCNL may be required due to early contra- or ipsilateral stone recurrence, especially in metabolic stone disease patients, regrowth of residual fragments, or planned staged procedures, all of which impose a burden on the patient and healthcare system [6–9].

Analyses of Canadian population-based data from 1991 to 2010, and of US population-based data from 2004 to 2013, and 2007 to 2017, have shown substantial increases in the use of URS with commensurate decreases in the use of SWL, whereas the use of PCNL has remained relatively stable [6, 10, 11]. The extent to which broad trends in the relative use of USSD procedures applies specifically to subsequent USSD procedures following PCNL, however, remains unknown. The purpose of this study was to describe trends and patterns of initial PCNL and subsequent USSD procedures from 2010 to 2019 among commercially insured US adults with USSD.

## Methods

### Study design and data source

This was a retrospective cohort study design with a 1-year baseline and up-to 3-year follow-up period anchored to an index date (defined as the first treatment with PCNL) using IBM^®^ MarketScan^®^ Commercial Claims and Encounters Database. The MarketScan^®^ database is a geographically diverse database consisting of inpatient, outpatient, and pharmacy insurance claims data for approximately 43.6 million active US employees and their dependents aged 64 or younger. This database captures the continuum of care covered by health insurance, providing longitudinal information on patients’ demographics and enrollment, physician office and other outpatient visits, hospital stays, and prescription drugs. This study was exempt from Institutional Review Board oversight because it contains de-identified patient records, as dictated by Title 45 Code of Federal Regulations (45 CFR 46, 101(b)(4)).

### Study population

The study population included adults between ages 18 and 64 years with USSD treated by PCNL between 01 January 2010 and 31 December 2019. Treatment with PCNL was identified by Current Procedural Terminology (CPT) codes (‘50,080’, ‘50,081’). Presence of USSD was confirmed on index date or in the baseline period using International Classification of Diseases, Ninth or Tenth Revision, Clinical Modification (ICD-9-CM ‘592.0’, ‘592.1’, ‘592.9’; ICD-10-CM ‘N20.0’, ‘N20.1’, ‘N20.2’, ‘N20.9’) codes. Patients were also required to have continuous insurance enrollment throughout the 12-month baseline period prior to the index date. Exclusion criteria included presence of stones in the lower urinary tract or presence of kidney or bladder tumors.

### Measures

#### Measurement of trends and patterns

Measures of interest for analysis of trends and patterns included the setting of initial PCNL (inpatient vs. outpatient), 1 vs. 2-step percutaneous access for the initial PCNL (percutaneous access performed at least one day before PCNL, usually by the radiologist), and the incidence, time course, and type of subsequent USSD procedures (SWL, PCNL, or URS) performed up-to 3 years after initial PCNL. A s*ubsequent USSD procedure* was defined as treatment by SWL (CPT code: ‘50,590’; Procedure codes: ICD-9 ‘98.51’, ICD-10 ‘0TF3XZZ’, ‘0TF4XZZ’), PCNL, and/or URS (CPT codes: ‘52,352’, ‘52,353’, ‘52,356’) with an accompanying anesthesia code (PCNL/URS only) during the 3-year follow-up period. Time-to the first subsequent USSD procedure was calculated as number of days from index PCNL to either the first USSD surgical intervention, end of continuous enrollment, or end of the study period, whichever came first. Information on stone type (e.g., struvite, cysteine, calcium oxalate monohydrate), laterality, or planned staged procedures was unavailable in the database and therefore subsequent USSD procedures are for any cause.

#### Measurement of patient characteristics

The baseline (12-month pre-index) period was used to measure patient characteristics for descriptive purposes. USSD-specific characteristics of interest included whether the patient had a prior USSD treatment (presence of SWL/URS CPT codes in the 12-month baseline period), location of stone (kidney, ureter, or both) and stone size (up to 2 cm, greater than 2 cm). Patient demographics and general clinical characteristics included age (18–34, 35–44, 45–54, 55–64 years), sex (female/male), insurance type (health maintenance organization [HMO], preferred provider organization [PPO], Other), Charlson Comorbidity Index Score (0, 1–2, 3–4, 5 and greater), Elixhauser comorbidities, prior urinary tract infection, geographic regions (Northeast, North Central, South, West, Unknown), and year of index PCNL (2010 through 2019).

### Statistical analyses

Descriptive statistics were presented as mean and standard deviation (SD) for continuous variables and as frequency and percentage for categorical variables. Kaplan–Meier curves depicting the incidence and time course of subsequent USSD procedures over the 3-year follow-up were created for all patients and for select sub-groups of interest. Log-rank tests were conducted to test for differences between the sub-groups of interest. Tests for trends in measures of interest by year of index PCNL were conducted using bivariable generalized linear models and Cox proportional hazards models. In a post-hoc analysis, we also conducted a multivariable logistic regression model to examine the association of patient and PCNL characteristics with undergoing PCNL in the outpatient vs. inpatient setting. A *P*-value ≤ 0.05 was considered statistically significant. All analyses were performed using StataSE 16 (StataCorp, College Station, Texas, US).

## Results

### Patient characteristics

A total of 8348 adult patients undergoing PCNL between 2010 and 2019 were eligible for the study. Supplemental Table 1 displays patient characteristics. Patients had a mean age of 50.6 (SD = 10.4) years and 54.5% were female. The three most prevalent diagnosed comorbidities were hypertension (55.2%), diabetes (33.0%), and obesity (20.4%). Overall, 25.2% had undergone either SWL (14.8%) or URS (14.6%) during the 12-month baseline period prior to PCNL; 4.2% had undergone both. Most patients (78.8%) were treated for stones in the kidney, and most initial PCNL procedures (70.0%) were directed at stones > 2 cm.

### Trends and patterns of setting and steps of initial PCNL

**Fig. 1:**
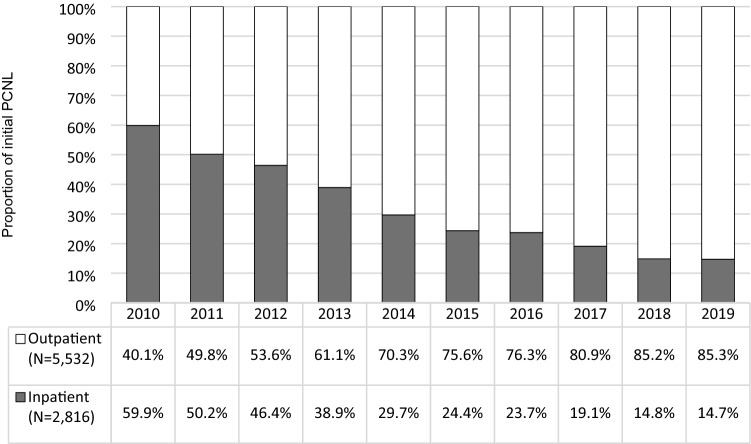
Proportion of initial PCNL performed in the inpatient vs. outpatient setting*. *P < 0.001 for trend from 2010 to 2019

Figure 1 shows the proportion of initial PCNL performed in the inpatient vs. outpatient setting by year. During the study period, there was a substantial shift in the setting of initial PCNL, from 59.9% being inpatient in 2010 to 85.3% being outpatient by 2019 (*P* < 0.001). Of the 5,532 initial PCNL performed in an outpatient setting, 134 (2.4%) were performed in an ambulatory surgical center.

Supplemental Table 2 shows the proportion of initial PCNL performed as a 1-step vs. 2-step procedure, for patients overall and by inpatient vs. outpatient setting for initial PCNL. The proportion of 1-step vs. 2-step initial PCNL fluctuated over time, with a low of 15.1% in 2016 and a high of 22.0% in 2019 but showed no consistent yearly trend (*P* = 0.137). Across all years, patients undergoing initial PCNL on the outpatient setting were more likely to have the procedure performed as a 2-step procedure than those undergoing initial PCNL on the inpatient setting (20.6% vs. 12.7%, *P* < 0.001); this pattern was also evident within each individual year (Supplemental Table 2).

### Trends and patterns of subsequent procedures after initial PCNL

**Fig. 2:**
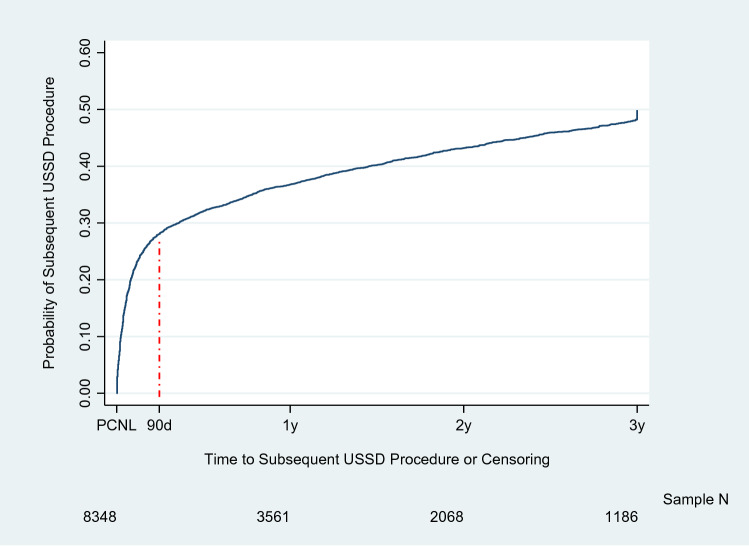
Kaplan–Meier estimated time course and probability of undergoing a subsequent USSD procedure

Over a mean follow-up of 13.5 (SD = 13.2) months, a total of 3,231 (39%) underwent a subsequent USSD procedure. Figure 2 shows the Kaplan–Meier estimated time course and probability of undergoing a subsequent USSD procedure for up-to 3 years following initial PCNL, accounting for censoring: 20.2% at 30 days, 25.6% at 60 days, 28.1% at 90 days, 32.0% at 180 days, 36.8% at 1 year, and 49.9% at 3 years. There was only a slight trend in the hazard of retreatment with increasing year (hazard ratio = 1.01, 95% confidence interval 1.0–1.02, *P* = 0.050).

Supplemental Fig. 1 shows the proportion of subsequent USSD procedures accounted for by SWL, PCNL, or URS by year of initial PCNL. From 2010 to 2019, the proportion of subsequent USSD procedures accounted for by URS increased substantially (from 30.8 to 51.8%), whereas SWL decreased substantially (from 39.5 to 14.7%) (*P* < 0.001).

Supplemental Table 3 displays the proportion subsequent USSD procedures accounted for by SWL, PCNL, or URS by year of initial PCNL and timing relative to initial PCNL: within 90 days, from 91 to 365 days, or from 1 to 3 years. Patients undergoing subsequent USSD procedures within 90 days were more likely to receive PCNL than SWL; however, this trend was reversed from 91 to 365 days, and then resumed from 1 to 3 years.

Supplemental Table 4 displays the results of the post-hoc analysis of factors associated with outpatient vs. inpatient PCNL. Older age, being male, more recent index year, and 2-step PCNL were significantly associated with greater odds of outpatient PCNL, whereas increasing Charlson Comorbidity Index score and having stones in both the kidney and ureter were associated with lower odds; having stones ≤ 2 cm was a marginally significant predictor (*P* = 0.051) of greater odds, and variation by region and health plan type was also observed.

## Discussion

Using data from 2010 to 2019, we studied trends and patterns of initial PCNL and subsequent USSD procedures among commercially-insured US adults with USSD. We observed several salient trends and patterns during the study period. First, a substantial shift has occurred in the setting of initial PCNL from the inpatient to the outpatient setting, with nearly 9 in 10 initial PCNLs being performed on the outpatient setting in 2019. Historically an inpatient procedure, technological advances have allowed for PCNL to be conducted in an outpatient setting, which is consistent with a growing trend of surgical procedures shifting to outpatient settings in an effort to reduce healthcare costs while maintaining patient safety [12, 13]. Although patients undergoing initial PCNL on the outpatient setting were more likely to receive a 2-step procedure than those undergoing initial PCNL on the inpatient setting, we did not observe any specific trend in the use of 2-step procedures within each setting. Future research examining whether outpatient performed PCNL is associated with differing risks of complications or need for subsequent USSD procedures in comparison with inpatient PCNL would be valuable.

Second, we observed a high rate of subsequent USSD procedures after initial PCNL: at 90 days, nearly one-third of patients had a subsequent USSD procedure, while by three years, this was the case for nearly half of patients. These findings are generally in line with previous studies reporting repeat surgeries after PCNL ranging from 38 to 45% over 1- and 5-year follow-up respectively [7–9]. Although we were unable to delineate the nature of subsequent procedures (stone recurrence, new stones formation on the opposite laterality, or planned secondary procedures), the persistent high rate of retreatment across the 10-year study period unfortunately does not suggest significant advancement in stone free rates and elimination of residual fragments, assuming no underlying systematic change in the complexity of cases over time. Approximately 56% (28%/50%) of subsequent procedures occurred within the first 90 days following the index PCNL, suggesting that a substantial proportion of subsequent procedures occurred in a timeframe during which stone formation after being stone free is an unlikely cause of need for a subsequent procedure, an indication that many patients were not stone free during this period [7].

Third, in line with prior research showing that URS has overtaken SWL as the most common initial USSD procedure, URS has also overtaken SWL as the most common subsequent procedure after initial PCNL [6, 10, 11, 14, 15]. The pattern of subsequent procedure choice also varied in relation to the time since initial PCNL, particularly in later years, with PCNL being far more likely to be used than SWL among patients requiring a subsequent procedure within 90 days (40% PCNL vs. 13% SWL in 2019), with the opposite being true for 91–365 days (18% PCNL vs. 24% SWL in 2019). This finding may be partially driven by planned secondary procedures in the first 90 days after initial PCNL, though PCNL resumed as the preferred choice from 1–2 years (37% PCNL vs. 15% SWL in 2019). Overall, by 2019, URS was always the preferred choice for subsequent USSD treatment.

Additional limitations, beyond not knowing the specific causes necessitating subsequent USSD procedures, included our inability to determine whether mini-PCNL, ultra-mini PCNL, or other variations of PCNL technology were used. Introduction and a subsequent update to the Centers for Medicare and Medicaid Services’ two-midnight rule for inpatient vs. outpatient classification, in 2013 and 2015, respectively, may have influenced some of the study findings, though we generally observed a smooth trend in the shift from inpatient to outpatient. This study focused specifically on commercially-insured individuals under the age of 65 and is therefore not necessarily generalizable to Medicare-insured patients and non-US populations. Finally, information on stone type (e.g., struvite, cysteine, calcium oxalate monohydrate) is not available in administrative claims data.

## Conclusion

From 2010 to 2019, PCNL procedures largely shifted to the outpatient setting. Subsequent procedures after initial PCNL were common, with most occurring within 90 days, indicating most patients were not completely stone-free after initial procedure. The continued climb in the incidence of subsequent treatments beyond 90 days confirms the long-term burden of USSD borne by patients. The rare possibility of metabolic rapid stone formers cannot simply explain a 90-day high rate of post-PCNL procedures. Whether for residual fragment(s) or part of a staged management, additional procedures following PCNL unveil an opportunity for improving primary true stone free status and/or offering safe single stage procedures, which seems a condition to reduce the 50% 3-year incidence of subsequent USSD procedure in the present study.

## Supplementary Information

Below is the link to the electronic supplementary material.

Supplementary file1 (DOCX 48 KB)

## Data Availability

The the IBM® MarketScan® Database is publicly available for commercial license. The authors of this study are not permitted to directly share the data.
