# Supplementary material for: Trends and patterns of initial percutaneous nephrolithotomy and subsequent procedures among commercially-insured US adults with urinary system stone disease: a 10-year population-based study
Source: World J Urol. 2022 Nov 19;41(1):235–40. doi: 10.1007/s00345-022-04210-0 (PMC9849290; doi:10.1007/s00345-022-04210-0)
Supplement: Supplementary file 1 — Supplementary file1 (DOCX 48 KB) [file 345_2022_4210_MOESM1_ESM.docx]

**Supplemental Figure 1. Proportion subsequent USSD procedures accounted for by SWL, PCNL, or URS***

**P<0.001 for trend from 2010—2019*

**Abbreviations**: SWL, extracorporeal shockwave lithotripsy; PCNL, Percutaneous nephrolithotomy; URS, ureteroscopy with laser lithotripsy; USSD, Urinary system stone disease

**Supplemental Table 1. Patient characteristics**

| **Variables** | | **Overall** | | | **Subsequent USSD Procedure** | | | | | | | |
| --- | --- | --- | --- | --- | --- | --- | --- | --- | --- | --- | --- | --- |
|  |  |  |  |  | **Yes** | | | | **No** | | | |
|  |  | **N** | | **%** | **N** | | **%** | | **N** | | | **%** |
| **ALL** | | **8,348** | | **100%** | **3,231** | | **39%** | | **5,117** | | | **61%** |
| **Age,** mean, SD | | 50.6 | | 10.4 | 50.0 | | 10.6 | | 51.0 | | | 10.3 |
| **Age category** | |  | |  |  | |  | |  | | |  |
| 18 to 34 years | | 767 | | 9.2 | 337 | | 10.4 | | 430 | | | 8.4 |
| 35 to 44 years | | 1,306 | | 15.6 | 516 | | 16.0 | | 790 | | | 15.4 |
| 45 to 54 years | | 2,636 | | 31.6 | 1,041 | | 32.2 | | 1,595 | | | 31.2 |
| 55 to 64 years | | 3,639 | | 43.6 | 1,337 | | 41.4 | | 2,302 | | | 45.0 |
| **Female** | | 4,553 | | 54.5 | 1,840 | | 56.9 | | 2,713 | | | 53.0 |
| **Insurance Plan Type** | |  | |  |  | |  | |  | | |  |
| HMO | | 786 | | 9.4 | 306 | | 9.5 | | 480 | | | 9.4 |
| PPO | | 5,077 | | 60.8 | 1,974 | | 61.1 | | 3,103 | | | 60.6 |
| Other | | 2,485 | | 29.8 | 951 | | 29.4 | | 1,534 | | | 30.0 |
| **CCI score** | |  | |  |  | |  | |  | | |  |
| 0 | | 4,176 | | 50.0 | 1,617 | | 50.0 | | 2,559 | | | 50.0 |
| 1 to 2 | | 3,104 | | 37.2 | 1,219 | | 37.7 | | 1,885 | | | 36.8 |
| 3 to 4 | | 798 | | 9.6 | 297 | | 9.2 | | 501 | | | 9.8 |
| 5 and greater | | 270 | | 3.2 | 98 | | 3.0 | | 172 | | | 3.4 |
| **Elixhauser comorbidities** | |  | |  |  | |  | |  | | |  |
| Congestive heart failure | | 222 | | 2.7 | 72 | | 2.2 | | 150 | | | 2.9 |
| Cardiac arrhythmias | | 1,009 | | 12.1 | 372 | | 11.5 | | 637 | | | 12.4 |
| Valvular disease | | 348 | | 4.2 | 130 | | 4.0 | | 218 | | | 4.3 |
| Pulmonary circulation disorders | | 108 | | 1.3 | 30 | | 0.9 | | 78 | | | 1.5 |
| Peripheral vascular disorders | | 303 | | 3.6 | 98 | | 3.0 | | 205 | | | 4.0 |
| Hypertension, uncomplicated | | 4,199 | | 50.3 | 1,624 | | 50.3 | | 2,575 | | | 50.3 |
| Hypertension, complicated | | 410 | | 4.9 | 156 | | 4.8 | | 254 | | | 5.0 |
| Paralysis | | 115 | | 1.4 | 44 | | 1.4 | | 71 | | | 1.4 |
| Other neurological disorders | | 253 | | 3.0 | 109 | | 3.4 | | 144 | | | 2.8 |
| Chronic pulmonary disease | | 1,024 | | 12.3 | 409 | | 12.7 | | 615 | | | 12.0 |
| Diabetes, uncomplicated | | 2,015 | | 24.1 | 786 | | 24.3 | | 1,229 | | | 24.0 |
| Diabetes, complicated | | 743 | | 8.9 | 268 | | 8.3 | | 475 | | | 9.3 |
| Hypothyroidism | | 988 | | 11.8 | 388 | | 12.0 | | 600 | | | 11.7 |
| Renal failure | | 656 | | 7.9 | 280 | | 8.7 | | 376 | | | 7.3 |
| Liver disease | | 900 | | 10.8 | 320 | | 9.9 | | 580 | | | 11.3 |
| Peptic ulcer disease | | 72 | | 0.9 | 24 | | 0.7 | | 48 | | | 0.9 |
| AIDS/HIV | | 25 | | 0.3 | 7 | | 0.2 | | 18 | | | 0.4 |
| Lymphoma | | 37 | | 0.4 | 12 | | 0.4 | | 25 | | | 0.5 |
| Metastatic cancer | | 48 | | 0.6 | 18 | | 0.6 | | 30 | | | 0.6 |
| Solid tumor without metastasis | | 390 | | 4.7 | 141 | | 4.4 | | 249 | | | 4.9 |
| Rheumatoid arthritis | | 318 | | 3.8 | 115 | | 3.6 | | 203 | | | 4.0 |
| Coagulopathy | | 213 | | 2.6 | 86 | | 2.7 | | 127 | | | 2.5 |
| Obesity | | 1,703 | | 20.4 | 644 | | 19.9 | | 1,059 | | | 20.7 |
| Weight loss | | 187 | | 2.2 | 71 | | 2.2 | | 116 | | | 2.3 |
| Fluid and electrolyte disorders | | 904 | | 10.8 | 385 | | 11.9 | | 519 | | | 10.1 |
| Blood loss anemia | | 80 | | 1.0 | 34 | | 1.1 | | 46 | | | 0.9 |
| Deficiency anemia | | 429 | | 5.1 | 168 | | 5.2 | | 261 | | | 5.1 |
| Alcohol abuse | | 79 | | 0.9 | 21 | | 0.6 | | 58 | | | 1.1 |
| Drug abuse | | 107 | | 1.3 | 36 | | 1.1 | | 71 | | | 1.4 |
| Psychoses | | 51 | | 0.6 | 19 | | 0.6 | | 32 | | | 0.6 |
| Depression | | 1,170 | | 14.0 | 430 | | 13.3 | | 740 | | | 14.5 |
| Urinary tract infection | | 378 | | 4.5 | 157 | | 4.9 | | 221 | | | 4.3 |
| **Regions** | |  | |  |  | |  | |  | | |  |
| Northeast | | 1,415 | | 17.0 | 489 | | 15.1 | | 926 | | | 18.1 |
| North Central | | 2,310 | | 27.7 | 828 | | 25.6 | | 1,482 | | | 29.0 |
| South | | 3,747 | | 44.9 | 1,563 | | 48.4 | | 2,184 | | | 42.7 |
| West | | 758 | | 9.1 | 298 | | 9.2 | | 460 | | | 9.0 |
| Unknown | | 118 | | 1.4 | 53 | | 1.6 | | 65 | | | 1.3 |
| **Index year** | |  | |  |  | |  | |  | | |  |
| 2010 | | 750 | | 9.0 | 266 | | 8.2 | | 484 | | | 9.5 |
| 2011 | | 939 | | 11.2 | 350 | | 10.8 | | 589 | | | 11.5 |
| 2012 | | 1,147 | | 13.7 | 428 | | 13.2 | | 719 | | | 14.1 |
| 2013 | | 971 | | 11.6 | 395 | | 12.2 | | 576 | | | 11.3 |
| 2014 | | 984 | | 11.8 | 395 | | 12.2 | | 589 | | | 11.5 |
| 2015 | | 784 | | 9.4 | 329 | | 10.2 | | 455 | | | 8.9 |
| 2016 | | 717 | | 8.6 | 317 | | 9.8 | | 400 | | | 7.8 |
| 2017 | | 669 | | 8.0 | 272 | | 8.4 | | 397 | | | 7.8 |
| 2018 | | 687 | | 8.2 | 261 | | 8.1 | | 426 | | | 8.3 |
| 2019 | | 700 | | 8.4 | 218 | | 6.7 | | 482 | | | 9.4 |
| **Prior SWL** | | 1,237 | | 14.8 | 459 | | 15.3 | | 742 | | | 14.5 |
| **Prior URS** | | 1,218 | | 14.6 | 554 | | 17.2 | | 664 | | | 13.0 |
| **Location of Stone** |  | |  | | |  | |  | |  |  | |
| Kidney | 6,576 | | 78.8 | | | 2,561 | | 79.3 | | 4,015 | 78.5 | |
| Ureter | 50 | | 0.6 | | | 20 | | 0.6 | | 30 | 0.6 | |
| Both | 1,722 | | 20.6 | | | 650 | | 20.1 | | 1,072 | 20.9 | |
| **Stone Size** |  | |  | | |  | |  | |  |  | |
| Up to 2cm | 2,502 | | 30.0 | | | 894 | | 27.7 | | 1,608 | 31.4 | |
| Greater than 2cm | 5,846 | | 70.0 | | | 2,337 | | 72.3 | | 3,509 | 68.6 | |

**Abbreviations**: AIDS, Acquired Immune Deficiency Syndrome; CCI, Charlson Comorbidity Index; SWL, extracorporeal shockwave lithotripsy; HIV, Human immunodeficiency virus; HMO, Health Maintenance Organization; PCNL, Percutaneous nephrolithotomy; PPO, Preferred Provider Organization; SD, Standard deviation; URS, ureteroscopy with laser lithotripsy; USSD, Urinary system stone disease

**Supplemental Table 2. Proportion of initial PCNL performed as a 1-step vs. 2-step procedure***

|  | **Overall (N=8,343)** | | **Inpatient (N=2,816)** | | **Outpatient (N=5,532)** | |
| --- | --- | --- | --- | --- | --- | --- |
| **Year** | 1-step | 2-step | 1-step | 2-step | 1-step | 2-step |
| **2010** | 84.9% | 15.1% | 90.4% | 9.6% | 76.7% | 23.3% |
| **2011** | 81.7% | 18.3% | 85.8% | 14.2% | 77.6% | 22.4% |
| **2012** | 80.0% | 20.0% | 85.7% | 14.3% | 75.1% | 24.9% |
| **2013** | 82.7% | 17.3% | 85.7% | 14.3% | 80.8% | 19.2% |
| **2014** | 82.9% | 17.1% | 88.4% | 11.6% | 80.6% | 19.4% |
| **2015** | 82.7% | 17.3% | 88.0% | 12.0% | 80.9% | 19.1% |
| **2016** | 84.9% | 15.1% | 90.0% | 10.0% | 83.4% | 16.6% |
| **2017** | 80.0% | 20.0% | 83.6% | 16.4% | 79.1% | 20.9% |
| **2018** | 83.1% | 16.9% | 87.3% | 12.7% | 82.4% | 17.6% |
| **2019** | 78.0% | 22.0% | 90.3% | 9.7% | 75.9% | 24.1% |
| ***P for trend*** | *P*=0.137 | | *P*=0.989 | | *P*=0.256 | |

**2-step defined as percutaneous access performed at least one day before PCNL*

**Supplemental Table 3. Proportion of subsequent USSD procedures accounted for by SWL, PCNL, or URS by year of initial PCNL and timing relative to initial PCNL**

|  | **0 – 90 days (N=2,285)** | | | **91 – 365 days (N=547)** | | | **1 year – 3 years (N=399)** | | |
| --- | --- | --- | --- | --- | --- | --- | --- | --- | --- |
|  | **PCNL** | **SWL** | **URS** | **PCNL** | **SWL** | **URS** | **PCNL** | **SWL** | **URS** |
| **2010** | 42% | 33% | 29% | 16% | 54% | 34% | 33% | 39% | 31% |
| **2011** | 43% | 26% | 36% | 9% | 52% | 40% | 33% | 33% | 38% |
| **2012** | 43% | 24% | 39% | 13% | 50% | 37% | 35% | 29% | 40% |
| **2013** | 47% | 27% | 32% | 15% | 42% | 42% | 37% | 30% | 37% |
| **2014** | 51% | 19% | 31% | 11% | 52% | 39% | 42% | 25% | 34% |
| **2015** | 45% | 20% | 40% | 16% | 54% | 34% | 35% | 28% | 41% |
| **2016** | 47% | 14% | 41% | 15% | 38% | 50% | 38% | 19% | 46% |
| **2017** | 39% | 15% | 50% | 11% | 38% | 51% | 32% | 20% | 52% |
| **2018** | 37% | 21% | 50% | 14% | 36% | 50% | 30% | 23% | 52% |
| **2019** | 40% | 13% | 51% | 18% | 24% | 58% | 37% | 15% | 52% |

**Supplemental Table 4. Multivariable logistic regression of the association of patient and PCNL characteristics with receipt of outpatient (outcome = 1) vs. inpatient (outcome = 0) PCNL**

| **N = 8,348** | **Odds Ratio** | **95% Confidence Interval** | | **P-value** |
| --- | --- | --- | --- | --- |
| **Age category** |  |  |  |  |
| 18 to 34 years | Reference | | | |
| 35 to 44 years | 1.15 | 0.95 | 1.41 | 0.160 |
| 45 to 54 years | 1.21 | 1.01 | 1.45 | 0.036 |
| 55 to 64 years | 1.26 | 1.06 | 1.51 | 0.010 |
| **Male** | 1.14 | 1.04 | 1.26 | 0.008 |
| **Insurance Plan Type** |  |  |  |  |
| Other | Reference | | | |
| HMO | 0.99 | 0.82 | 1.19 | 0.874 |
| PPO | 0.86 | 0.77 | 0.96 | 0.008 |
| **CCI score** |  |  |  |  |
| 0 | Reference | | | |
| 1 to 2 | 0.93 | 0.84 | 1.04 | 0.197 |
| 3 to 4 | 0.79 | 0.67 | 0.94 | 0.009 |
| 5 and greater | 0.68 | 0.51 | 0.89 | 0.006 |
| **Regions** |  |  |  |  |
| Northeast | Reference | | | |
| North Central | 1.58 | 1.37 | 1.83 | <0.001 |
| South | 1.80 | 1.58 | 2.07 | <0.001 |
| West | 1.03 | 0.85 | 1.25 | 0.769 |
| Unknown | 6.38 | 3.78 | 10.76 | <0.001 |
| **Index year** | 1.30 | 1.28 | 1.33 | <0.001 |
| **Prior SWL** | 1.14 | 0.99 | 1.31 | 0.069 |
| **Prior URS** | 1.11 | 0.96 | 1.28 | 0.153 |
| **Location of Stone** |  |  |  |  |
| Both | Reference | | | |
| Kidney | 1.43 | 1.27 | 1.61 | <0.001 |
| Ureter | 2.61 | 1.31 | 5.19 | 0.006 |
| **Stone Size** |  |  |  |  |
| Up to 2cm | Reference | | | |
| Greater than 2cm | 1.11 | 1.00 | 1.24 | 0.051 |
| **PCNL steps** |  |  |  |  |
| 1-step | Reference | | | |
| 2-steps | 1.94 | 1.69 | 2.23 | <0.001 |

**Abbreviations**: CCI, Charlson Comorbidity Index; SWL, extracorporeal shockwave lithotripsy; HMO, Health Maintenance Organization; PCNL, Percutaneous nephrolithotomy; PPO, Preferred Provider Organization; URS, ureteroscopy with laser lithotripsy
